# The relationships between race, employment, and self-rated health among older South Africans: exploring the mediating role of generalized anxiety

**DOI:** 10.3389/fpubh.2024.1398705

**Published:** 2024-11-20

**Authors:** Adams Yunus, Lulin Zhou, Seidu Abdulai Jamatutu, Evelyn Agba Tackie

**Affiliations:** ^1^School of Management, Jiangsu University, Zhenjiang, Jiangsu, China; ^2^School of Economics and Management, Nanjing University of Science and Technology, Nanjing, Jiangsu, China

**Keywords:** employment, anxiety, older adults, retirement, South Africa

## Abstract

**Objective:**

This study explores the impact of race and employment status on self-rated health among older adults in South Africa. It reveals new insights by examining the mediating role of generalized anxiety. The findings highlight the importance of addressing these factors to improve the wellbeing of older adults.

**Methods:**

Drawing from Wave 5 of the National Income Dynamics Study—Coronavirus Rapid Mobile Survey, a cross-sectional survey encompassing a nationally representative cohort of South African adults, this research employs a sophisticated blend of logistic regression and structural equation modeling techniques.

**Results:**

The study reveals that race is strongly linked to self-rated health. Individuals of Colored and White backgrounds have lower odds of favorable self-rated health. Retirement has a positive impact on self-rated health. Generalized anxiety mediates the complex relationship between race and self-rated health.

**Conclusions:**

Addressing the imperative need to mitigate racial disparities in self-rated health and advance equitable healthcare access for older adults, targeted interventions are undeniably warranted.

## 1 Introduction

In South Africa and globally, the proportion of older adults is growing substantially (1). Their health and wellbeing are pivotal, particularly considering economic disparities (2). This study delves into the complex relationship between race, employment status, generalized anxiety, and self-rated health (SRH) among older South African adults. By examining the interaction between race and employment status and the mediating role of generalized anxiety, this research seeks to understand the combined effects of these variables on SRH.

Like many other countries, South Africa's geriatric population confronts distinct health challenges (3). Notably, older South Africans face a higher prevalence of chronic diseases, disabilities, and mental health conditions compared to their younger counterparts (4). Factors such as sociodemographic characteristics and healthcare access contribute to these disparities (5), with similar patterns observed globally (6).

Recognizing the health challenges of older adults, both state and non-state actors have initiated programs and policies to promote healthy aging, enhance healthcare accessibility, and bolster social support (7, 8). This study acknowledges these advancements and emphasizes the need for further refinement of these strategies.

This research focuses on the relationship between self-rated health, race, employment status, and generalized anxiety in older individuals (9). Race plays a significant role in health disparities due to South Africa's history of apartheid (10). Employment status affects access to resources and overall wellbeing (11). Generalized anxiety is a prevalent mental health condition in older individuals (12).

Studies on self-rated health among South African older adults show its significance in predicting mortality, morbidity, and quality of life (9). It is associated with socioeconomic factors, healthcare access, and wellbeing. Certain racial groups report lower self-rated health (13), which may be attributed to historical racial inequality (14). Work-related stress and retirement have implications for self-rated health (15).

The current literature reveals that generalized anxiety may have a considerable impact on self-rated health in older individuals (16). It may be a potential mediator in the relationship between race, employment, and self-rated health (16). Generalized anxiety may arise due to various stressors related to race-based experiences and employment-related factors, influencing individuals' perceptions of their health (16). Exploring the combined impact of race and employment status on self-rated health in older South Africans is essential to understanding potential interactive effects.

Previous research has investigated the relationships between race, employment status, and generalized anxiety and the SRH of older adults (17–20). These studies have revealed that race influences health outcomes through historical and contemporary experiences, whereas employment status influences socioeconomic status and access to healthcare. In addition, it has been demonstrated that generalized anxiety, a prevalent mental health condition among older adults, has negative impacts on overall health (16). However, the combined impact of race and employment status on SRH, as well as the potential mediating role of generalized anxiety in this relationship, remain largely unexplored.

This study examines the relationships between race, employment status, generalized anxiety, and self-rated health among older adults using data from the National Income Dynamics study—Coronavirus Rapid Mobile Survey. It employs robust statistical methods to provide a comprehensive understanding of their intricate interplay. The findings will inform targeted interventions and strategies that promote the wellbeing of older adults.

## 2 Materials and methods

We analyzed the Wave 5 data from the NIDS-CRAM survey, which studies the socioeconomic effects of South Africa's 2020 State of Disaster declaration and national lockdown (21). The survey involves a Computer-Assisted Telephone Interviewing approach and covers income, employment, household welfare, grant receipt, and COVID-19-related knowledge and behaviors. Our analysis focused on 1,453 respondents aged 50 and above. We obtained informed consent from all participants and conducted our research in accordance with the Declaration of Helsinki. The University of Cape Town Commerce Ethics Committee approved (REC 2020/04/017) and ensured reciprocal ethics compliance from Stellenbosch University.

### 2.1. Measures

We collected data on participants' self-rated health, prompting them to rate their health as excellent, very good, good, fair, or poor. We collected data on race/population groups: African/Black, Colored, Asian/Indian, and White. To gauge generalized anxiety levels, we employed the GAD-2 instrument, consisting of two questions. The first question inquired, “Over the last 2 weeks, have you had little interest in doing things?” while the second asked, “Over the last 2 weeks, have you been feeling down, depressed or hopeless?” Scores on the GAD-2 scale range from 0 to 6, where higher scores signify elevated anxiety levels. Our analysis integrates various sociodemographic variables: age, gender, marital status, education, household income, employment status, dwelling type, and residence.

### 2.2. Theoretical framework and rationale

Our research framework builds on the Theory of Social Determinants of Health (22) and the Biopsychosocial Model (23), which emphasize the influence of sociodemographic attributes and multiple factors on health outcomes. We acknowledge the role of generalized anxiety, as assessed through the GAD-2 scale, in the association between race/employment status and self-rated health. Our framework also embraces Intersectionality Theory (24), recognizing the interplay of multiple social identities contributing to health disparities. We consider employment status an independent variable, in line with the Job Strain Theory (25), which suggests job demands and control can affect stress levels and health perceptions.

### 2.3. Statistical analysis

We used STATA SE version 14.2 and Intellectus Statistics to analyze the data. Descriptive statistics were used to summarize variables and explore their relationship with self-rated health via binary logistic regression. We then employed structural equation modeling (SEM) to investigate the role of generalized anxiety (GAD-2) as a mediator in the relationship between race, employment status, and self-rated health. We performed path analysis to ensure the regression model accurately describes the data. For this analysis, we used two regression formulas. The first formula determined the role of generalized anxiety as a moderator between race and self-rated health. The second formula investigated the mediating role of generalized anxiety in the relationship between employment status and self-rated health.

## 3 Results

Table 1 provides an overview of socioeconomic and demographic variables. The majority of the population falls under the 50–59 age group, followed by the 60–69, 70–79, and 80+ age categories. Females make up 70.5% of respondents. African/Black individuals form the majority, followed by Colored, White, and Asian/Indian individuals. Most individuals are married and reside in houses. Grade 12 and Grade 7 have the highest educational attainment. Household income mainly falls between R1,500 and R5,000. Unemployed individuals form the majority, followed by retired and employed individuals. Self-rated health is distributed among different categories, with good health being the highest proportion.

**Table 1:** Descriptive statistics of the study variables.

**Variable**	**Proportion**	**Std. Err**.	**95% confidence**	**Interval**
**Age**				
50–59	0.443	0.030	0.384	0.503
60–69	0.303	0.028	0.251	0.360
70–79	0.177	0.023	0.136	0.228
80 +	0.077	0.016	0.051	0.116
**Gender**				
Male	0.295	0.028	0.244	0.353
Female	0.705	0.028	0.647	0.756
**Race**				
African/Black	0.727	0.027	0.670	0.777
Colored	0.155	0.022	0.116	0.203
Asian/Indian	0.004	0.004	0.001	0.026
White	0.114	0.019	0.081	0.158
**Marital status**				
Married	0.723	0.027	0.667	0.774
Single	0.277	0.027	0.226	0.333
**Residence**				
Traditional area/chiefdom	0.114	0.019	0.081	0.158
Urban area/town	0.395	0.030	0.338	0.455
Farm/rural area	0.491	0.030	0.431	0.551
**Dwelling type**				
A house	0.768	0.026	0.713	0.814
Traditional house like a	0.173	0.023	0.133	0.224
An informal house like	0.037	0.011	0.020	0.067
Other	0.022	0.009	0.010	0.049
**Education level**				
Grade R/0	0.031	0.005	0.023	0.041
Grade 1 (previously Sub A/class 1)	0.025	0.004	0.018	0.034
Grade 2 (previously Sub B/class 2)	0.033	0.005	0.025	0.044
Grade 3 (Std. 1)	0.032	0.005	0.024	0.042
Grade 4 (Std. 2)	0.049	0.006	0.039	0.061
Grade 5 (Std. 3)	0.054	0.006	0.044	0.067
Grade 6 (Std. 4)	0.061	0.006	0.049	0.074
Grade 7 (Std. 5)	0.108	0.008	0.093	0.125
Grade 8 (Std. 6/form 1)	0.090	0.008	0.076	0.106
Grade 9 (Std. 7/form 2)	0.051	0.006	0.041	0.064
Grade 10 (Std. 8/form 3)	0.090	0.008	0.076	0.106
Grade 11 (Std. 9/form 4)	0.061	0.006	0.050	0.075
Grade 12 (Std10/Matric/Senior Certificate)	0.211	0.011	0.191	0.233
National certificate vocational 2	0.001	0.001	0.000	0.005
National certificate vocational 3	0.001	0.001	0.000	0.005
National certificate vocational 4	0.002	0.001	0.001	0.006
N1 (NATED)/NTC 1	0.001	0.001	0.000	0.005
N2 (NATED)/NTC 2	0.001	0.001	0.000	0.005
N3 (NATED)/NTC 3	0.002	0.001	0.001	0.006
No schooling	0.092	0.008	0.078	0.108
ABET level 1	0.001	0.001	0.000	0.005
ABET level 2	0.001	0.001	0.000	0.005
ABET level 3	0.003	0.001	0.001	0.007
**Household income**				
Less than R1,500	0.070	0.016	0.045	0.108
Between R1,500 and R3,000	0.439	0.030	0.381	0.499
Between R3,000 and R5,000	0.236	0.026	0.189	0.291
More than R5,000	0.255	0.027	0.206	0.310
**Employment status**				
Employed	0.196	0.024	0.152	0.247
Unemployed	0.480	0.030	0.420	0.540
Retired	0.325	0.028	0.271	0.383
**Self-rated health**				
Excellent	0.081	0.017	0.054	0.121
Very good	0.207	0.025	0.162	0.259
Good	0.339	0.029	0.285	0.398
Fair	0.214	0.025	0.169	0.267
Poor	0.159	0.022	0.120	0.207

Std. Err., standard error; Sub, substitute; Std., standard; Matric, matriculation; NTC, National Technical Certificate; ABET, Adult Basic Education Training.

The regression analysis in Table 2 shows the relationship between race, employment status, and self-rated health among older adults. The study indicates that White individuals had significantly lower odds of rating their health as good, while retired individuals had significantly higher odds in Model 1. However, no significant associations were observed for unemployment in any of the models. Moreover, age, gender, marital status, residence, dwelling type, education, household income, and generalized anxiety were not significantly associated with self-rated health.

**Table 2:** Association between self-rated health and race, employment status, and their interactions.

**Variable**	**Self-rated health**		
	**Model 1**	**Model 2**	**Model 3**
	**OR (95% CI)**	**OR (95% CI)**	**OR (95% CI)**
**Race**			
African/Black	Ref	Ref	Ref
Colored	0.34 (0.21; 0.56)^***^	0.21 (0.91; 0.49)^***^	0.16 (0.01; 3.41)
Asian/Indian	0.29 (0.10; 0.81)^**^	0.64 (0.07; 5.45)	0.31 (0.02; 3.85)
White	0.26 (0.16; 0.43)^***^	0.16 (0.07; 0.34)^***^	0.02 (0.001; 0.25)^**^
**Employment status**			
Employed	Ref	Ref	Ref
Unemployed	1.34 (0.87; 2.06)	1.07 (0.60; 1.91)	0.51 (0.05; 5.01)
Retired	1.95 (1.23; 3.11)^**^	1.25 (0.65; 2.43)	0.51 (0.01; 0.25)
**Interactions**			
Colored #Unemployed		1.76 (0.52; 5.95)	2.45 (0.05; 7.61)
Colored #Retired		2.53 (0.73; 8.74)	1.13 (0.02; 5.67)
Asian/Indian #Unemployed		0.31 (0.02; 3.90)	0.41 (0.05; 4.23)
Asian/Indian #Retired		0.19 (0.01; 5.08)	1.19 (0.69; 8.22)
White #Unemployed		1.81 (0.51; 6.42)	2.44 (0.21; 6.58)
White #Retired		2.90 (0.93; 8.99)	9.09 (0.34; 11.17)
Age			1.18 (0.62; 2.26)
Gender			0.32 (0.07; 1.37)
Marital status			0.47 (0.14; 1.50)
Recidence			0.49 (0.17; 1.37)
Dwelling type			0.48 (0.23; 0.97)
Education			0.97 (0.87; 1.07)
Household income			0.66 (0.36; 1.23)
Generalized anxiety			3.52 (0.65; 6.34)

OR, odds ratio; CI, confidence interval; #, interaction.

^***^p <0.001.

^**^p <0.01.

**Figure 1:**
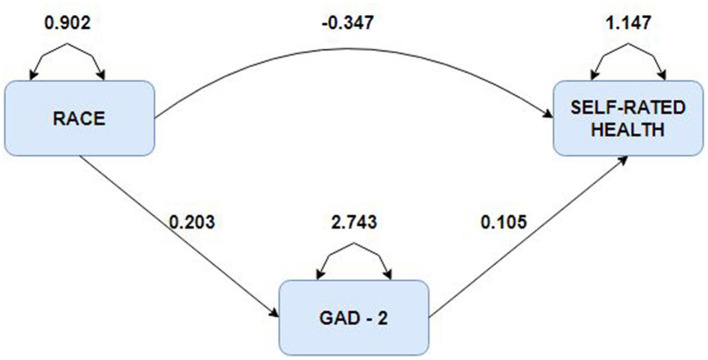
Diagram of the mediating role of generalized anxiety in the relationship between race and self-rated health.

In addition, a mediation analysis was conducted to investigate the role of generalized anxiety (GAD-2) as a mediator between race and self-rated health (SRH) among older adults. The analysis's reliability was gauged based on the sample size used to construct the model. The model's path analysis results are shown in Table 3, while Figure 1 illustrates the node diagram for the mediating role of GAD-2 in the association between race and SRH.

**Table 3:** Path analysis [The role of generalized anxiety (GAD-2) as a mediator between race and self-rated health].

**Parameter estimate**	**Unstandardized**	**Standardized**	***p***
**Regressions**			
Race → SRH	−0.35 (0.03)	−0.29	<0.001
Race → GAD-2	0.20 (0.05)	0.12	<0.001
GAD-2 → SRH	0.11 (0.02)	0.16	<0.001
Indirect effect of race on SRH through GAD-2	0.02 (0.006)	0.02	<0.001
Total effect of race on SRH	−0.33 (0.03)	−0.27	<0.001
**Errors**			
Error in race	0.90 (0.03)	1.00	<0.001
Error in GAD-2	2.74 (0.10)	0.99	<0.001
Error in SRH	1.15 (0.04)	0.90	<0.001

SRH, self-rated health; GAD-2, generalized anxiety; p, significance level.

The study included 1,453 participants and six variables with a participant-to-item ratio of 242 to 1. Results showed that race significantly predicted both SRH and GAD-2. GAD-2 partially mediated the relationship between race and SRH, indicating that an increase in race would increase the SRH value by 0.02 units. The total effect of a one-unit increase in race resulted in a decrease in SRH by 0.33 units.

**Figure 2:**
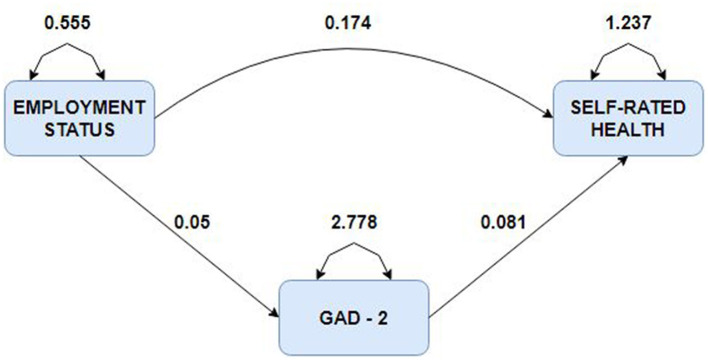
Diagram of the mediating role of generalized anxiety in the relationship between employment status and self-rated health.

The role of generalized anxiety (GAD-2) as a mediator between employment status and self-rated health (SRH) was investigated. Table 4 displays the results of the path analysis, while Figure 2 provides a node diagram illustrating GAD-2's mediating role.

**Table 4:** Path analysis [The role of generalized anxiety (GAD-2) as a mediator between employment status and self-rated health].

**Parameter estimate**	**Unstandardized**	**Standardized**	***p***
**Regressions**			
Employment status → GAD-2	0.05 (0.06)	0.02	0.391
GAD-2 → SRH	0.08 (0.02)	0.12	<0.001
Employment status → SRH	0.17 (0.04)	0.11	<0.001
Indirect effect of employment on SRH through GAD-2	0.004 (0.005)	0.003	0.399
Total effect of employment on SRH	0.18 (0.04)	0.12	<0.001
**Errors**			
Error in employment status	0.56 (0.02)	1.00	<0.001
Error in GAD-2	2.78 (0.10)	1.00	<0.001
Error in SRH	1.24 (0.05)	0.97	<0.001

SRH, self-rated health; GAD-2, Generalized Anxiety Disorder-2 item; p, significance level.

Employment didn't significantly predict GAD-2, but GAD-2 significantly predicted SRH. An increase in GAD-2 by one unit is expected to increase SRH by 0.08 units. Similarly, an increase in Employment by one unit increases the expected SRH value by 0.17 units. Mediation analysis showed that GAD-2 doesn't fully mediate the relationship between Employment and SRH, but partial mediation might exist. The indirect effect of Employment on SRH through GAD-2 was not significant, but the total effect of Employment on SRH was significant. This implies that GAD-2 doesn't support partial mediation in this context.

## 4 Discussion

This study investigated the relationships between race, employment status, self-rated health (SRH), and generalized anxiety in older adults and the mediating role of generalized anxiety in these relationships. Significant associations and interactions were found between these variables, revealing a complex interplay.

The binary logistic regression results showed significant associations between race and self-rated health (SRH) among older adults. Colored individuals had lower odds of rating their health as good, while White older individuals also reported lower odds of self-rated health. Multiple factors may explain these associations (13). In the case of Colored individuals, socioeconomic disparities, limited access to healthcare, or cultural factors that influence their perception of health may contribute to lower SRH probabilities (13). Similarly, the initial lower odds in Model 1 for Asian/Indian individuals may indicate cultural or socioeconomic influences on SRH, which may become less significant when controlling for additional variables in Models 2 and 3 (26). On the other hand, White individuals had consistently lower odds of self-reported excellent health across all models, further validating the effect of race on health perceptions (27).

White older adults may have lower odds due to resource access, discrimination, or health behavior differences (27). Prior research has consistently shown that race can significantly impact self-rated health (27, 28). Retirees were significantly associated with good health in the first model but not in subsequent models. Unemployment did not directly affect self-rated health in this study population. Unemployment's impact on self-rated health may be influenced by financial stability, social support, or individual coping techniques (29). Due to various factors, retirement status is significantly associated with higher odds of self-rated excellent health in Model 1 (30). Retirement may improve one's health by reducing work stress, increasing leisure time, and providing better access to healthcare and social support (30). Unlike prior studies (31), which showed lower self-rated health among the unemployed, these findings call for further investigations into contextual factors influencing this relationship in South Africa.

No significant associations were found between race, employment status and self-rated health for the interaction terms. This indicates that the combined effect of race and employment status on self-rated health in this study population is not significant. However, it is important to note that the absence of significant interactions does not imply the absence of complex relationships; therefore, additional research is required to investigate potential interaction effects in various contexts.

Generalized anxiety (GAD-2) partially mediates the influence of race on self-rated health (SRH), indicating that it may play a role in explaining the relationship between race and SRH. Racial discrimination, social inequalities, and cultural factors may lead to higher levels of generalized anxiety, which in turn affects health perception. However, there is no significant mediation effect between employment status and SRH, suggesting that job satisfaction, social support, and working conditions may be more important in explaining this relationship among older adults.

In this analysis, age, gender, marital status, residence, dwelling type, education and household income were not significantly associated with self-rated health (SRH). Still, previous research indicates that these sociodemographic factors are essential in determining health perceptions (32, 33). For example, age is usually linked with health deterioration associated with accumulated life stresses and the onset of chronic diseases, thus lowering self-rated health in older people (34). SRH is well documented to be different by gender, with women often reporting lower SRH than men, perhaps because of greater exposure to life stressors and chronic illness, as well as differences in healthcare utilization and health perceptions (35).

Another factor that may be related to SRH is marital status because married people are relatively more likely to report good health than unmarried people, possibly due to the social support marriage offers to most of them (36). In addition, SRH may be differently affected by residence and dwelling type because, for example, they are generally associated with better health outcomes through access to health services and supportive housing conditions (37). Well-recognized determinants of health are education and household income, measured by socioeconomic status, and education and income levels tend to reflect improvements in SRH where higher education and income have positive effects on health through mechanisms such as resource availability, health-promoting lifestyles, and health literacy (38).

However, these variables did not show significant associations in this study, but further research might further explain the intricate interactions between sociodemographic factors and older adults' SRH.

This study shows that race and employment status significantly predict the self-rated health of older adults in South Africa. Addressing racial disparities and employment conditions is crucial to improving their wellbeing, along with targeted interventions to reduce health inequalities and improve healthcare access. Policymakers should adopt a comprehensive approach that considers employment opportunities and socioeconomic factors to support the health and wellbeing of older adults.

The study's strengths lie in its use of a nationally representative survey, a validated measure of generalized anxiety, and structural equation modeling. However, its cross-sectional design limits causal interpretations, self-rated health outcome measures may be biased, and self-rated data may induce recall and reporting biases.

## 5 Conclusion

This study found that Colored and White individuals in South Africa have a lower likelihood of reporting excellent self-rated health. Retirement was associated with increased odds of self-rated excellent health. Generalized anxiety partially mediates the relationship between race and self-rated health. Policymakers should address racial health disparities and promote equitable access to healthcare services. Further research is needed to investigate the complex interactions between race, employment status, and health outcomes.

## Data Availability

Publicly available datasets were analyzed in this study. This data can be found at: https://cramsurvey.org/reports/.
